# Anti-EGFR enhanced neoadjuvant immunotherapy versus neoadjuvant immunochemotherapy for locally advanced oral squamous cell carcinoma

**DOI:** 10.3389/fimmu.2025.1669368

**Published:** 2025-11-20

**Authors:** Peng Li, Qiufeng Jin, Mingwei Su, Qigen Fang, Wei Du

**Affiliations:** Department of Head Neck and Thyroid, The Affiliated Cancer Hospital of Zhengzhou University & Henan Cancer Hospital, Zhengzhou, China

**Keywords:** neoadjuvant immunotherapy, cetuximab, neoadjuvant chemotherapy, oral squamous cell carcinoma, prognosis

## Abstract

**Background:**

Immune checkpoint inhibitors have transformed the management of metastatic oral squamous cell carcinoma (OSCC), but their efficacy in the neoadjuvant setting is still under investigation. This study aimed to compare the efficacy and safety of neoadjuvant immunochemotherapy (NAIC), neoadjuvant immunotherapy plus cetuximab (NAI-CTX), and neoadjuvant chemotherapy (NAC) in patients with locally advanced OSCC.

**Methods:**

In this retrospective cohort study, 475 patients with stage III-IVA OSCC were stratified into three groups: NAIC (n=265), NAI-CTX (n=46), and NAC (n=164). The primary endpoint was pathologic complete response (pCR). Secondary endpoints included event-free survival (EFS), overall survival (OS), major pathologic response (mPR), and treatment-related toxicity.

**Results:**

Pathologic outcomes were significantly superior in the NAIC group. The pCR rates were 30.2% for NAIC, compared to 13.0% for NAI-CTX and 7.3% for NAC (p<0.001). Similarly, mPR rates were 69.8%, 39.1%, and 50.0%, respectively (p<0.001). NAIC also achieved a higher objective response rate (90.6% *vs.* 71.7% *vs.* 65.9%, p<0.001) and a near-complete R0 resection rate (98.1% *vs.* 89.1% *vs.* 97.0%). Survival analysis revealed 3-year EFS rates of 73.6% (NAIC), 56.5% (NAI-CTX), and 46.3% (NAC) (p<0.0001), with corresponding 3-year OS rates of 78.1%, 63.0%, and 59.1% (p<0.0001). Multivariable analysis confirmed the independent survival benefit of NAIC. Toxicity profiles differed: the NAI-CTX group exhibited more cetuximab-related toxicities, while NAIC was associated with a higher incidence of hypothyroidism. No treatment-related deaths occurred. The efficacy of neoadjuvant therapy was primarily associated with PD-L1 expression levels rather than the number of treatment cycles.

**Conclusion:**

NAIC demonstrates superior pathologic responses and survival benefits compared to both NAI-CTX and NAC, establishing it as a highly promising neoadjuvant strategy for locally advanced OSCC.

## Introduction

Head and neck squamous cell carcinoma (HNSCC), particularly oral squamous cell carcinoma (OSCC), remains a therapeutic challenge in locally advanced stages, with 5-year survival rates stagnating at 40–60% despite multimodal therapy (1). The advent of immunotherapy, specifically immune checkpoint inhibitors (ICIs) targeting PD-1/PD-L1, has revolutionized systemic treatment for recurrent/metastatic disease (1, 2). However, the role of ICIs in the neoadjuvant setting for resectable OSCC is still evolving, with recent trials demonstrating promising pathologic response rates but inconsistent survival benefits (3–5).

Neoadjuvant immunotherapy leverages the pre-surgical window to prime antitumor immunity, potentially eradicating micrometastases and improving long-term outcomes. Studies such as the phase II trial by Vos et al. (nivolumab + ipilimumab) reported major pathologic responses (MPR) in 35% of HNSCC patients, including OSCC, with correlative biomarker data suggesting immune activation (2). Similarly, Wu et al. demonstrated a 42% MPR rate with neoadjuvant camrelizumab (anti-PD-1) combined with chemotherapy, underscoring the synergy between ICIs and cytotoxic agents (4). Yet, the optimal combination strategy—balancing efficacy, toxicity, and biomarker selection—remains unresolved (6, 7).

A critical gap lies in the potential of EGFR-targeted agents to enhance ICI efficacy. Cetuximab, an anti-EGFR monoclonal antibody, has immunomodulatory properties, including NK cell activation and dendritic cell maturation, which may complement PD-1 blockade (8). While neoadjuvant immunochemotherapy (e.g., PD-1 inhibitor + platinum/5-FU) is under investigation (4, 9), no studies have directly compared this approach to immunotherapy + Cetuximab in OSCC. Preliminary data from Goldfarb et al. in lacrimal duct carcinomas suggest EGFR inhibition may improve pathologic responses when combined with ICIs (9), but evidence in OSCC is lacking.

Therefore, this retrospective cohort study was designed to directly compare the efficacy and safety of three neoadjuvant strategies [neoadjuvant immunochemotherapy (NAIC) *vs.* neoadjuvant immunotherapy plus cetuximab (NAI-CTX) *vs.* neoadjuvant chemotherapy (NAC)] in patients with locally advanced, resectable OSCC. Our primary objective was to determine whether NAI-CTX yields pathologic and survival outcomes that are superior, comparable, or inferior to those achieved with NAIC or NAC. A secondary objective was to identify potential clinicopathological and molecular biomarkers, particularly PD-L1 expression, associated with treatment response. By addressing these questions, we aim to provide evidence to guide the selection of optimal neoadjuvant regimens in this patient population.

## Patients and methods

### Ethical approval

This study was approved by Henan Cancer Hospital Institutional Research Committee, and written informed consent for medical research was obtained from all patients before starting the treatment. All methods were performed in accordance with the relevant guidelines and regulations.

### Study design

We performed a retrospective cohort study analyzing consecutive patients with locally advanced OSCC who received neoadjuvant therapy followed by curative-intent surgery at a tertiary cancer between January 2015 and December 2024. The study population comprised two contemporary treatment groups receiving immunotherapy-based regimens and one historical control group treated with conventional chemotherapy. Eligible patients met the following criteria: histologically confirmed, previously untreated OSCC (stage III-IVA per AJCC 8th edition); completion of planned neoadjuvant therapy; availability of complete clinicopathological data. We excluded patients with: prior history of malignancy (except non-melanoma skin cancers) within 5 years; palliative surgery; insufficient follow-up data (<6 months unless recurrence/death occurred earlier).

The patient enrollment process was detailed in **Supplementary Figure S1**. Briefly, from an initial pool of 765 patients with OSCC, 365 were treated between January 2015 and June 2019, and 400 were treated between July 2019 and December 2024. Patients with early-stage disease and those who did not receive neoadjuvant therapy were excluded. This resulted in 278 eligible patients with locally advanced OSCC in the earlier period, who constituted the NAC group. From the later period, 333 eligible OSCC patients received neoadjuvant immunotherapy and were stratified into the NAIC (n=265) and NAI-CTX (n=46) groups based on their treatment regimen, forming the final study cohort of 475 patients.

The number of neoadjuvant cycles was determined by the treating multidisciplinary tumor board based on individual patient factors, including treatment tolerance, early radiographic response assessed after 2 cycles, and the feasibility of timely surgery. Patients who exhibited significant toxicity or achieved sufficient downstaging after 2 cycles typically proceeded to surgery, while those with ongoing good tolerance and potential for further response received a third cycle.

The contemporary cohort was divided into two groups based on neoadjuvant regimen from July 2019 to December 2024. In NAIC group, patients received combination therapy with an anti-PD-1 inhibitor (pembrolizumab or tislelizumab or penpulimab 200 mg IV every 3 weeks) plus platinum-based chemotherapy (cisplatin 75mg/m² and docetaxel 75mg/m²) for two or three cycles. In NAI-CTX group, patients underwent 2 or 3 cycle anti-PD-1 therapy (same dosing as NAIC group) combined with cetuximab (400 mg/m² loading dose followed by 250 mg/m² weekly). The historical NAC group consisted of patients treated between January 2015 and June 2019 who received standard neoadjuvant chemotherapy (cisplatin 75 mg/m² and docetaxel 75mg/m²) for two or three cycles.

Trained research personnel abstracted data from electronic medical records using standardized case report forms. Collected variables included demographic characteristics, clinical staging, treatment details, pathological staging, recurrence patterns, and survival outcomes.

### Variable definition

Clinical staging (cTNM) was determined through comprehensive pretreatment evaluation according to the American Joint Committee on Cancer Cancer Staging Manual, 8th edition. Pathological staging (ypTNM) was assessed by two independent pathologists evaluating surgical specimens following neoadjuvant therapy according to AJCC guidelines for post-treatment classification. Histopathological grading employed the WHO classification system for OSCC, categorizing tumors as well differentiated, moderately differentiated, and poorly differentiated. PD-L1 expression was quantitatively assessed using the 22C3 pharmDx assay (Agilent Technologies, Santa Clara, CA) on pretreatment biopsy specimens. The combined positive score (CPS) was calculated as the number of PD-L1-staining cells (tumor cells, lymphocytes, macrophages) divided by the total number of viable tumor cells, multiplied by 100.

The primary endpoint was pCR, defined as the absence of viable tumor cells (ypT0) and nodal metastases (ypN0) in surgical specimens following neoadjuvant therapy, as confirmed by two pathologists using standardized histopathologic protocols (10). Secondary endpoints included major pathologic response (mPR; ≤10% viable tumor cells) (10), R0 resection rate (microscopically negative margins ≥1 mm), objective response rate (ORR) assessed per RECIST 1.1 criteria (11) through serial contrast-enhanced CT/MRI, with complete response (CR) and partial response (PR) constituting responses, event-free survival (EFS), overall survival (OS), and treatment-related toxicity.

EFS was defined as the time interval from the initiation of neoadjuvant therapy to the first occurrence of any of the following events: histologically confirmed local or regional recurrence following curative-intent surgery, development of distant metastases, or death from any cause; patients who did not experience any of these events were censored at the date of their last documented disease evaluation. OS was calculated from the first day of neoadjuvant treatment until death from any cause, with living patients censored at their last known follow-up date. Treatment-emergent adverse events were recorded during the neoadjuvant treatment period and for 30 days following treatment completion, graded according to the Common Terminology Criteria for Adverse Events (CTCAE) version 5.0 (12). Toxicity was categorized by severity: minor toxicity (Grade 1-2 events not requiring treatment discontinuation) and major toxicity (Grade 3-4 events necessitating dose modification or therapy interruption).

### Treatment principles

The extent of surgical resection was determined on a case-by-case basis through a multidisciplinary team discussion following neoadjuvant therapy. The decision to base the resection on the initial tumor extent or the post-neoadjuvant downsized extent was individualized, balancing the paramount goal of oncologic safety with the objectives of functional preservation and postoperative quality of life. Key factors influencing this decision included the degree of radiographic and clinical response, the feasibility and complexity of the planned reconstruction, the anatomical subsite involved, and the patient’s overall performance status and preferences. For the neck, a comprehensive dissection of the levels initially involved was typically performed. This holistic and patient-tailored strategy ensured that the high R0 resection rate was achieved while actively considering the functional and cosmetic outcomes for each patient.

Adjuvant treatment strategies were determined through multidisciplinary tumor board consensus, incorporating comprehensive evaluation of both preoperative imaging features and postoperative pathologic findings. Postoperative radiotherapy (PORT) commenced within six weeks following surgery, precisely targeting the tumor bed with a margin of 1-2 cm and delivering a prescribed dosage of 60-66 Gy. The postoperative chemoradiotherapy (POCRT) employed a platinum-based regimen consisting of 4-6 cycles. Following treatment completion, patients will undergo close monitoring with clinical and radiologic evaluations every 3 months during the first year, then every 3-6 months in the second year. Subsequent follow-ups will occur every 6 months through year 5 to ensure timely detection of potential recurrence.

### Statistical analysis

For baseline characteristics, continuous variables were presented as means with standard deviations or medians with interquartile ranges, depending on distribution normality assessed by Shapiro-Wilk tests. Categorical variables were reported as frequencies and percentages. Between-group comparisons used ANOVA or Kruskal-Wallis tests for continuous variables and χ² or Fisher’s exact tests for categorical variables, as appropriate.

pCR was compared between groups using multivariable logistic regression adjusting for clinically relevant covariates including age, sex, clinical cancer stage, tumor differentiation. Secondary endpoints were analyzed similarly: mPR followed the same analytical approach as pCR, while radiologic responses and R0 resection were compared using χ² tests.

Survival outcomes were analyzed with rigorous time-to-event methods. EFS and OS curves were generated using the Kaplan-Meier method and compared with log-rank tests. Cox proportional hazards models were constructed to adjust for potential confounding variables, including margin status, extracapsular extension, and adjuvant therapy receipt. The proportional hazards assumption was verified using Schoenfeld residuals. Treatment-related toxicity analyses compared major adverse event rates between groups using χ² tests.

For missing data (present in <5% of cases for most variables), we employed multiple imputation using chained equations (5 imputed datasets). Sensitivity analyses using complete cases only showed consistent results.

All statistical tests were two-sided, with a significance threshold of p < 0.05. Analyses were performed using R software (version 4.3.1; R Foundation for Statistical Computing) and SPSS (version 27.0; IBM Corp).

## Results

### Baseline data

In total, 475 patients with locally advanced OSCC were enrolled and stratified into three treatment groups: NAIC (n=265), NAI-CTX (n=46), and NAC (n=164). Baseline characteristics were balanced across groups, though the NAI-CTX cohort had a significantly higher proportion of elderly patients (>60 years: 60.9% *vs.* 35.8% in NAIC and 45.1% in NAC, P = 0.032). Most patients were male (72.0%), smokers (54.9%), and had stage IV disease (56.8%), with no significant differences in sex, smoking status, primary tumor site, or PD-L1 CPS distribution among groups (all P>0.05). Pathologic outcomes varied markedly by treatment: pCR (yp0) rates were highest in the NAIC group (30.2%), followed by NAI-CTX (13.0%) and NAC (7.3%) (P<0.001). Similarly, advanced ypStage (III-IV) was less frequent in NAIC (15.1%) compared to NAI-CTX (39.1%) and NAC (51.2%), reflecting superior downstaging with immunochemotherapy (**Supplementary Table S1**).

### pCR

NAIC demonstrated superior efficacy in achieving pCR compared to other regimens, with a pCR rate of 30.2% *versus* 13.0% for NAI-CTX and 7.3% for NAC (p < 0.001). Multivariable analysis confirmed NAIC as the strongest predictor of pCR (reference, OR = 1.00), while both NAC (OR = 0.19, p < 0.001) and NAI-CTX (OR = 0.38, p = 0.008) were significantly less effective. Tumor characteristics played a critical role, with PD-L1 CPS >20 tumors nearly tripling the likelihood of pCR (OR = 2.95, p < 0.001), whereas poorly differentiated histology (OR = 0.32, p < 0.001) and Stage IV disease (OR = 0.48, p = 0.002) were associated with poorer responses. Age >60 years also modestly reduced pCR rates (OR = 0.62, p = 0.022), but no significant associations were found for sex, smoking, alcohol use, the number of neoadjuvant therapy cycles, or tumor location. These results highlight the importance of both treatment selection and tumor biology in optimizing pathologic responses for locally advanced OSCC (**Table 1**).

**Table 1:** Predictors for pCR achievement in all the patients.

Variable	Logistic regression	Multivariable logistic regression	
	p	OR [95%CI]	p
Age			
≤60		ref	
>60	0.021	0.62 [0.41-0.93]	0.022
Sex			
Male			
Female	0.387		
Smoker			
No			
Yes	0.109		
Drinker			
No			
Yes	0.215		
Primary site			
Tongue	ref		
Mouth floor	0.452		
Buccal	0.287		
Gingiva	0.623		
Differentiation			
Well		ref	
Moderate	0.034	0.59 [0.37-0.95]	0.029
Poor	<0.001	0.32 [0.18-0.57]	<0.001
CPS^&^			
<1		ref	
1-20	0.018	1.82 [1.12-2.96]	0.016
>20	<0.001	2.95 [1.72-5.06]	<0.001
cTNM			
III		ref	
IV	0.003	0.48 [0.30-0.77]	0.002
Cycle			
Two			
Three	0.156		
Neoadjuvant therapy			
NAIC		ref	
NAI-CTX	0.008	0.38 [0.19-0.78]	0.008
NAC	<0.001	0.19 [0.10-0.36]	<0.001

^&^CPS, combined positive score.

### mPR

The highest rate of mPR was observed with NAIC (69.8%), followed by NAC (50.0%) and NAI-CTX (39.1%), with significant differences across groups (p < 0.001). Multivariable analysis reinforced NAIC as the most effective regimen (reference), with NAC (OR = 0.43, p < 0.001) and NAI-CTX (OR = 0.28, p < 0.001) showing substantially lower odds of mPR. PD-L1 CPS >20 remained a strong positive predictor (OR = 2.58, p < 0.001), while poorly differentiated tumors (OR = 0.35, p < 0.001) and Stage IV disease (OR = 0.55, p = 0.005) again correlated with worse outcomes. The effect of age >60 years was less pronounced for mPR than for pCR (OR = 0.67, p = 0.048). Importantly, the consistent treatment hierarchy (NAIC > NAC > NAI-CTX) and predictive value of PD-L1 across both endpoints suggest these factors are robust determinants of therapeutic success, supporting the potential for biomarker-guided treatment strategies in OSCC (**Table 2**).

**Table 2:** Predictors for mPR achievement in all the patients.

Variable	Logistic regression	Multivariable logistic regression	
	p	OR [95%CI]	p
Age			
≤60		ref	
>60	0.045	0.67 [0.45-0.99]	0.048
Sex			
Male			
Female	0.512		
Smoker			
No			
Yes	0.087		
Drinker			
No			
Yes	0.210		
Primary site			
Tongue			
Mouth floor	0.398		
Buccal	0.265		
Gingiva	0.715		
Differentiation			
Well		ref	
Moderate	0.028	0.62 [0.41-0.95]	0.026
Poor	<0.001	0.35 [0.21-0.58]	<0.001
CPS^&^			
<1		ref	
1-20	0.013	1.72 [1.12-2.64]	0.014
>20	<0.001	2.58 [1.54-4.32]	<0.001
cTNM			
III		ref	
IV	0.006	0.55 [0.36-0.84]	0.005
Cycle			
Two			
Three	0.290		
Neoadjuvant therapy			
NAIC		ref	
NAI-CTX	<0.001	0.28 [0.15-0.52]	<0.001
NAC	<0.001	0.43 [0.29-0.65]	<0.001

^&^CPS, combined positive score.

### R0 resection/ORR

Negative margins (R0 resection) were achieved in 460 patients (96.8% overall). The R0 resection rates varied significantly among treatment groups: 98.1% (260/265) in the NAIC group, 89.1% (41/46) in the NAI-CTX group, and 97.0% (159/164) in the NAC group (p = 0.012). In the NAIC cohort, CR and PR were achieved in 69 (26.0%) and 171 (64.5%) patients, respectively. In the NAI-CTX group, CR and PR rates were 3 (4.3%) and 30 (65.2%), while the NAC group had 9 (5.5%) CR and 99 (60.4%) PR. This resulted in ORR of 90.6% (NAIC), 71.7% (NAI-CTX), and 65.9% (NAC), with statistically significant differences across groups (p < 0.001).

### Adverse event

The majority of toxicities were Grade 1 or 2 in severity. The NAI-CTX group exhibited a distinct toxicity profile, characterized by significantly higher incidences of any-grade diarrhea (15.2% *vs.* 3.4% NAIC *vs.* 2.4% NAC, p<0.001) and rash (13.0% *vs.* 4.2% *vs.* 1.2%, p=0.001), consistent with the known effects of cetuximab. Hypothyroidism, an immune-related adverse event, was exclusively observed in the NAIC group (4.1%, p=0.007). Regarding severe (Grade 3/4) events, the overall incidence was low. The NAIC group had the highest rate of severe neutropenia (3.8%), though this was not statistically significant compared to other groups (p=0.121). Notably, the NAI-CTX group experienced a significantly higher incidence of severe rash (8.7%) compared to both the NAIC (0.4%) and NAC (0.0%) groups (p<0.001). No Grade 3/4 diarrhea, hypothyroidism, or pneumonitis was reported in the NAI-CTX group, and no treatment-related deaths occurred in any cohort (**Table 3**).

**Table 3:** Adverse events during neoadjuvant therapy among NAIC, NAI-CTX, and NAC groups.

Adverse event	NAIC (n=265)	NAI-CTX (n=46)	NAC (n=164)	p
Grade 1/2				
Neutropenia	38 (14.3%)	4 (8.7%)	20 (12.2%)	0.521
Anemia	18 (6.8%)	2 (4.3%)	10 (6.1%)	0.782
Thrombocytopenia	14 (5.3%)	3 (6.5%)	7 (4.3%)	0.722
Diarrhea	9 (3.4%)	7 (15.2%)	4 (2.4%)	<0.001
Rash	11 (4.2%)	6 (13.0%)	2 (1.2%)	0.001
Hypothyroidism	10 (3.7%)	0	0	0.007
Fatigue	19 (7.2%)	5 (10.9%)	14 (8.5%)	0.621
Hepatotoxicity	15 (5.7%)	4 (8.7%)	5 (3.0%)	0.142
Pneumonia	4 (1.5%)	0	1 (0.6%)	0.512
Mucositis	11 (4.2%)	3 (6.5%)	8 (4.9%)	0.732
Grade 3/4				
Neutropenia	10 (3.8%)	0	2 (1.2%)	0.121
Hypothyroidism	1 (0.4%)	0	0	0.642
Rash	1 (0.4%)	4 (8.7%)	0	<0.001

### EFS

**Figure 1:**
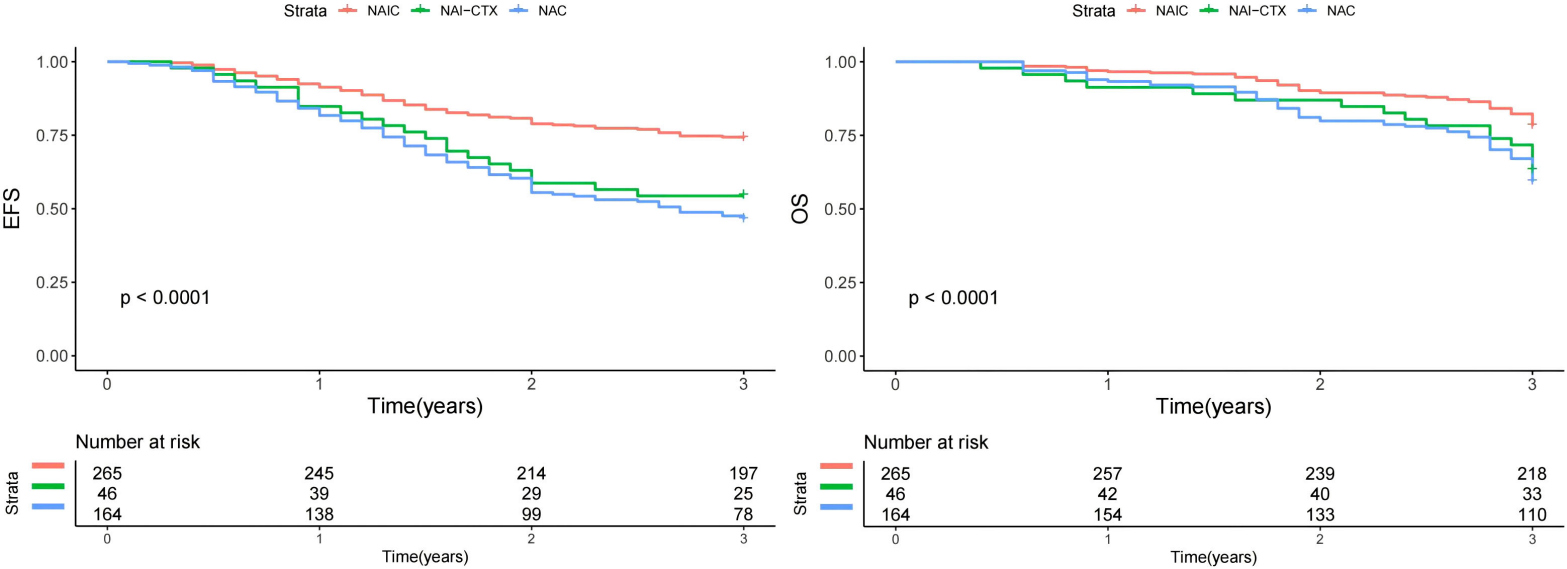
Comparison of 3-year event free survival (EFS) and overall survival (OS) in patients treated by neoadjuvant therapies.

During our follow-up with a median time of 40 months, there were 178 recurrences and 142 deaths. The 3-year EFS rates were 73.6%, 56.5%, and 46.3% in NAIC, NAI-CTX, and NAC groups, the difference was significant (p<0.0001, **Figure 1**). Poor differentiation (multivariable HR = 1.95, p=0.001) and Stage IV disease (HR = 1.70, p<0.001) were independently associated with worse EFS, while PD-L1 CPS >20 (HR = 0.55, p=0.003) predicted improved outcomes. NAIC demonstrated superior efficacy as the reference treatment, with both NAC (HR = 1.55, p=0.006) and NAI-CTX (HR = 1.40, p=0.064) showing higher relapse risks. Age, sex, and lifestyle factors (smoking/drinking) did not reach statistical significance in the multivariable model. These results underscore the importance of tumor biology and treatment selection in determining EFS (**Table 4**).

**Table 4:** Univariate and multivariable cox model analysis of predictors for event-free survival.

Variable	Univariate		Multivariable	
	HR [95%CI]	p	HR [95%CI]	p
Age (>60 vs ≤60)	1.32 [0.98-1.78]	0.067	1.25 [0.92-1.70]	0.152
Sex (Male vs female)	1.08 [0.79-1.47]	0.634		
Smoker (Yes vs no)	1.22 [0.91-1.64]	0.185		
Drinker (Yes vs no)	1.15 [0.85-1.56]	0.367		
Primary site				
Tongue	ref		ref	
Mouth floor	1.10 [0.75-1.62]	0.623	1.07 [0.72-1.59]	0.734
Buccal	1.25 [0.88-1.78]	0.210	1.20 [0.84-1.72]	0.316
Gingiva	1.40 [0.97-2.02]	0.072	1.35 [0.93-1.96]	0.116
Differentiation				
Well	ref		ref	
Moderate	1.45 [1.02-2.06]	0.038	1.40 [0.98-2.00]	0.063
Poor	2.10 [1.45-3.04]	<0.001	1.95 [1.34-2.85]	0.001
CPS^&^				
<1	ref		ref	
1-20	0.75 [0.52-1.08]	0.121	0.78 [0.54-1.13]	0.188
>20	0.52 [0.35-0.77]	0.001	0.55 [0.37-0.82]	0.003
cTNM (IV vs III)	1.80 [1.35-2.40]	<0.001	1.70 [1.27-2.28]	<0.001
Cycle (Three vs two)	1.34 [0.87-1.82]	0.367		
Neoadjuvant therapy				
NAIC	ref		ref	
NAI-CTX	1.45 [1.02-2.06]	0.039	1.40 [0.98-2.00]	0.064
NAC	1.60 [1.18-2.17]	0.002	1.55 [1.14-2.11]	0.006

^&^CPS, combined positive score.

### OS

For OS, there were 142 deaths, the 3-year OS rates were 78.1%, 63.0%, and 59.1% in NAIC, NAI-CTX, and NAC groups, the difference was significant (p<0.0001, **Figure 1**). Poor differentiation (HR = 2.15, p<0.001), and Stage IV disease (HR = 1.90, p<0.001) were negative predictors, whereas PD-L1 CPS >20 (HR = 0.48, p<0.001) remained strongly protective. NAIC again outperformed other regimens, with both NAC (HR = 1.70, p=0.002) and NAI-CTX (HR = 1.50, p=0.034) associated with increased mortality. The impact of age (>60 years) was marginal in multivariable analysis (HR = 1.35, p=0.067). The consistency of these findings with EFS analysis reinforces the critical roles of tumor differentiation, PD-L1 status, and neoadjuvant strategy in survival outcomes (**Table 5**).

**Table 5:** Univariate and multivariable cox model analysis of predictors for overall survival.

Variable	Univariate		Multivariable	
	HR [95%CI]	p	HR [95%CI]	p
Age (>60 vs ≤60)	1.40 [1.02-1.92]	0.037	1.35 [0.98-1.86]	0.067
Sex (Male vs female)	1.12 [0.80-1.56]	0.512		
Smoker (Yes vs no)	1.30 [0.95-1.78]	0.102		
Drinker (Yes vs no)	1.20 [0.87-1.65]	0.267		
Primary site				
Tongue	ref		ref	
Mouth floor	1.15 [0.76-1.74]	0.502	1.12 [0.74-1.70]	0.594
Buccal	1.35 [0.93-1.96]	0.116	1.30 [0.89-1.90]	0.178
Gingiva	1.55 [1.06-2.26]	0.024	1.50 [0.82-2.20]	0.239
Differentiation				
Well	ref		ref	
Moderate	1.60 [1.10-2.33]	0.015	1.55 [1.06-2.26]	0.024
Poor	2.30 [1.55-3.42]	<0.001	2.15 [1.45-3.20]	<0.001
CPS^&^				
<1	ref		ref	
1-20	0.70 [0.47-1.04]	0.079	0.72 [0.48-1.08]	0.112
>20	0.45 [0.30-0.68]	<0.001	0.48 [0.32-0.72]	<0.001
cTNM (IV vs III)	2.00 [1.46-2.74]	<0.001	1.90 [1.38-2.62]	<0.001
Cycle (Three vs two)	1.22 [0.56-1.99]	0.342		
Neoadjuvant therapy				
NAIC	ref		ref	
NAI-CTX	1.55 [1.07-2.25]	0.021	1.50 [1.03-2.18]	0.034
NAC	1.75 [1.26-2.43]	0.001	1.70 [1.22-2.37]	0.002

^&^CPS, combined positive score.

## Discussion

This study provides compelling evidence that NAIC represents a paradigm shift in the treatment of locally advanced OSCC, demonstrating superior efficacy across pathologic, surgical, and survival endpoints compared to both NAC and NAI-CTX. The NAIC regimen achieved remarkable pCR rates of 30.2% - more than quadruple that of NAC (7.3%) and double NAI-CTX (13.0%) - with corresponding improvements in mPR (69.8% *vs.* 39.1-50.0%) and R0 resection rates (98.1% *vs.* 89.1-97.0%). These pathologic benefits translated into clinically meaningful survival advantages, with NAIC patients experiencing 3-year EFS and OS rates of 73.6% and 78.1% respectively, representing absolute improvements of 17-27 percentage points over comparator arms. Importantly, while NAIC was associated with expected hematologic toxicity, it demonstrated a more favorable safety profile than NAI-CTX, which showed significantly higher rates of cetuximab-related adverse events. These findings position NAIC as a potential standard of care in neoadjuvant OSCC treatment.

NAC has been investigated as a strategy to improve outcomes in OSCC, yet its clinical utility remains constrained by several limitations (13). While NAC aims to downstage tumors and eradicate micrometastases, real-world data suggest inconsistent survival benefits. A landmark nationwide cohort study (14) analyzing 29,891 OSCC patients demonstrated equivalent 5-year disease-specific survival (DSS: 62% *vs.* 66%, p=0.116) and OS (56% *vs.* 57%, p=0.992) between NAC followed by surgery *versus* surgery alone cohorts after propensity matching. Notably, the study revealed paradoxical outcomes: patients with pT4a tumors actually fared worse with NAC (5-year DSS: 52% *vs.* 62%, p=0.0006), suggesting potential harm in advanced cases. However, responders achieving pCR showed exceptional outcomes (5-year DSS: 95% *vs.* 60%), highlighting the critical importance of patient selection. The TPF regimen (docetaxel-cisplatin-5-fluorouracil), while demonstrating superior pCR rates (20-30%) in hypopharyngeal/laryngeal SCC, has shown more modest efficacy in OSCC, with pCR rates typically below 15% in most series (15). This discrepancy may reflect fundamental biological differences between anatomical subsites, including distinct tumor microenvironments and HPV prevalence patterns. Several key challenges persist in NAC implementation: The lack of reliable predictive biomarkers beyond clinical staging makes patient selection empirical; Post-NAC treatment protocols remain heterogeneous, with no consensus on optimal adjuvant therapy for partial responders; Surgical planning becomes complicated by treatment-induced fibrosis and anatomical changes. Our institutional experience mirrors these limitations - among 164 OSCC patients receiving NAC between 2015-2019, only 7.3% achieved pCR, while 50% showed mPR. Importantly, we observed no significant improvement in 3-year EFS compared to upfront surgery (46.3% *vs.* 42.0%, p=0.412) (unpublished data). These findings underscore the need for paradigm-shifting approaches.

The established survival benefit of immunotherapy in recurrent/metastatic HNSCC (16) has driven significant interest in its neoadjuvant application, either as monotherapy or in combination regimens. The phase III KEYNOTE-689 trial (17) evaluated this approach in 714 locally advanced HNSCC patients randomized to pembrolizumab (two neoadjuvant cycles plus 15 adjuvant cycles) *versus* standard therapy (surgery ± PORT/POCRT). The trial demonstrated a significant improvement in 36-month EFS with pembrolizumab across all populations (CPS≥10: 59.8% *vs.* 45.9%, HR 0.66, p=0.004; CPS≥1: 58.2% *vs.* 44.9%, HR 0.70, p=0.003; overall: 57.6% *vs.* 46.4%, HR 0.73, p=0.008), establishing a clear benefit for event-free survival. However, the observed pathologic response rates were modest (mPR: 9.4% overall, 13.7% in CPS≥10), and at the time of this interim analysis, a statistically significant overall survival benefit had not yet emerged. This may suggest that while neoadjuvant and adjuvant anti-PD-1 therapy effectively enhances locoregional control, more potent regimens including those combining immunotherapy with cytotoxic chemotherapy could be required to induce deeper pathologic responses that more reliably translate into an OS advantage, particularly in a disease with a high risk of distant metastasis. Safety data revealed comparable Grade≥3 AE rates (44.6% *vs.* 42.9%) but higher immune-mediated toxicity with pembrolizumab (10.0% Grade≥3), including slightly increased fatal events (1.1% *vs.* 0.3%). These findings suggest that while anti-PD-1 therapy enhances locoregional control, its standalone use may be insufficient to overcome the systemic disease burden in HNSCC, underscoring the need for optimized combination strategies to improve pathologic responses and survival outcomes.

Emerging evidence suggests that combining neoadjuvant immunotherapy with chemotherapy may enhance treatment efficacy in HNSCC. A recent single-arm study (18) of 42 HNSCC patients treated with NAIC demonstrated a pCR rate of 33% (6/18) among those who underwent surgery. Similarly, Xiang et al. (19) reported even more promising results in their cohort of 31 patients, with 29 undergoing surgical resection. In this study, the mPR rate reached 69.0% (20/29, 95% CI: 49.2–84.7%), while the pCR rate was 41.4% (12/29, 95% CI: 23.5–61.1%). Notably, all three patients with stage IVB disease achieved mPR in both primary tumors and cervical lymph nodes, and among 26 patients with stage III/IVA disease, 17 (65.4%) achieved mPR in primary tumors (including 11 pCR) and 14 (53.8%) in lymph nodes (including 8 pCR). Radiographic assessment showed an ORR of 82.8% (24/29, 95% CI: 64.2–94.2%) by RECIST v1.1 criteria, with 7 CR and 17 PR. These findings are supported by a meta-analysis of 13 studies involving 458 NAIC-treated patients (20), which reported pooled mPR and pCR rates of 61% and 37%, respectively, along with a 91% disease-free survival rate. Importantly, patients with PD-L1 CPS ≥20 had 2.09-fold higher odds of achieving mPR compared to those with CPS <20 (AUC = 0.76 for radiographic prediction of mPR). While these results demonstrate the potential of NAIC to induce significant pathologic responses, important limitations remain. Cisplatin-based regimens require adequate renal function, and these trials often exclude elderly patients (≥70 years), highlighting the need for alternative treatment strategies for these subpopulations.

Cetuximab has demonstrated established efficacy in recurrent/metastatic HNSCC through both the TPEx and EXTREME regimens (21, 22). Its potential role in the neoadjuvant setting was preliminarily explored in a small study (N = 21) combining cetuximab with immunotherapy and chemotherapy (23), which reported promising outcomes: a 66.7% mPR rate including 11 pCR (52.4%), and an ORR of 90.5% with 28.6% CR. Notably, oropharyngeal tumors showed particular sensitivity to this approach. The regimen demonstrated a favorable safety profile, with anemia (61.9%) as the most common adverse event and no grade 4 toxicities or surgical delays reported. Impressively, laryngeal preservation was achieved in 90.9% of cases (10/11), with all patients attaining negative surgical margins. In our current study, we evaluated 46 patients receiving NAI-CTX. This cohort was notably older than our other treatment groups, reflecting our clinical preference for cetuximab over chemotherapy in elderly patients. While pathologic responses in this group surpassed those reported in KEYNOTE-689’s pembrolizumab monotherapy arm (17), they remained inferior to our NAIC cohort’s outcomes. These findings suggest that cetuximab may enhance the efficacy of immunotherapy alone while maintaining a manageable toxicity profile, positioning NAI-CTX as a viable alternative for chemotherapy-ineligible patients. However, the optimal patient selection criteria and long-term outcomes for this approach require further investigation.

In current clinical practice, the optimal duration of neoadjuvant immunotherapy for OSCC remains undefined, with most centers empirically administering two to three cycles in the absence of formal guidelines. Notably, no comparative studies have specifically evaluated the efficacy of two *versus* three cycles in OSCC. However, this question has been actively investigated in other solid tumors, yielding conflicting results. A recent study of 108 non-small cell lung cancer (NSCLC) patients (24) reported that two cycles (*versus* ≥3 cycles) were associated with smaller post-treatment tumor size (37.0 mm *vs.* 49.6 mm, p=0.022) and higher radiographic regression rates (36% *vs.* 49%, p=0.007), though pathological response rates were comparable between groups. Conversely, another NSCLC trial (N = 176) (25) demonstrated no significant correlation between cycle number (ranging from 2 to ≥5) and ORRs (52-67%), mPR, or pCR. Importantly, surgical outcomes—including operative time, postoperative drainage, and length of hospitalization—were unaffected by treatment duration. A meta-analysis (26) further confirmed that while neoadjuvant immunotherapy improved 2-year disease-free survival and pCR rates in NSCLC, these benefits were independent of treatment-related factors such as PD-L1 expression, platinum regimen, or number of cycles. Our findings align with this emerging consensus that neoadjuvant therapy efficacy appears largely independent of cycle number. This observation holds particular clinical relevance for OSCC management, where minimizing treatment burden without compromising outcomes is paramount. Based on available evidence, a two-cycle neoadjuvant therapy regimen may represent a balanced approach—offering comparable efficacy to longer courses while potentially reducing toxicity in vulnerable populations. However, prospective OSCC-specific studies are urgently needed to validate this approach and identify optimal patient selection criteria.

The limitations of current research must be acknowledged: firstly, there are inherent selective biases in retrospective studies; Secondly, our sample size of NAI-CTX is relatively small, which may reduce our statistical ability; Thirdly, external validation is required before clinical application.

In summary, this study highlights the transformative potential of NAIC in locally advanced OSCC, achieving unprecedented pCR and survival rates compared to NAI-CTX and NAC. The robust efficacy of NAIC, coupled with manageable toxicity, suggests it could redefine neoadjuvant standards, particularly for PD-L1-high tumors. While NAI-CTX offers an alternative for chemotherapy-ineligible patients, its inferior efficacy underscores the need for further biomarker refinement.

## Data Availability

The original contributions presented in the study are included in the article/**Supplementary Material**. Further inquiries can be directed to the corresponding author.

## Supplementary material

Supplementary Figure 1 Patient enrollment flowchart.
