# Supplementary material for: Anti-EGFR enhanced neoadjuvant immunotherapy versus neoadjuvant immunochemotherapy for locally advanced oral squamous cell carcinoma
Source: Front Immunol. 2025 Nov 20;16:1669368. doi: 10.3389/fimmu.2025.1669368 (PMC12675437; doi:10.3389/fimmu.2025.1669368)
Supplement: Supplementary file 1 [file Supplementaryfile1.doc]

Supplementary Table 1. Baseline data of all enrolled patients.

| Variable | Total  (n=475) | NAIC  (n=265) | NAI-CTX  (n=46) | NAC  (n=164) | P* |
| --- | --- | --- | --- | --- | --- |
| Age |  |  |  |  |  |
| ≤60 | 278 (58.5%) | 170 (64.2%) | 18 (39.1%) | 90 (54.9%) |  |
| >60 | 197 (41.5%) | 95 (35.8%) | 28 (60.9%) | 74 (45.1%) | 0.032 |
| Sex |  |  |  |  |  |
| Male | 343 (72.0%) | 190 (71.7%) | 35 (76.1%) | 117 (71.3%) |  |
| Female | 133 (28.0%) | 75 (28.3%) | 11 (23.9%) | 47 (28.7%) | 0.421 |
| Smoker |  |  |  |  |  |
| No | 214 (45.1%) | 125 (47.2%) | 18 (39.1%) | 71 (43.3%) |  |
| Yes | 261 (54.9%) | 140 (52.8%) | 28 (60.9%) | 93 (56.7%) | 0.215 |
| Drinker |  |  |  |  |  |
| No | 298 (62.7%) | 170 (64.2%) | 25 (54.3%) | 103 (62.8%) |  |
| Yes | 177 (37.3%) | 95 (35.8%) | 21 (45.7%) | 61 (37.2%) | 0.178 |
| Primary site |  |  |  |  |  |
| Tongue | 198 (41.7%) | 110 (41.5%) | 22 (47.8%) | 66 (40.2%) |  |
| Mouth floor | 112 (23.6%) | 65 (24.5%) | 9 (19.6%) | 38 (23.2%) |  |
| Buccal | 95 (20.0%) | 50 (18.9%) | 8 (17.4%) | 37 (22.6%) |  |
| Gingiva | 70 (14.7%) | 40 (15.1%) | 7 (15.2%) | 23 (14.0%) | 0.309 |
| Differentiation |  |  |  |  |  |
| Well | 142 (29.9%) | 80 (30.2%) | 12 (26.1%) | 50 (30.5%) |  |
| Moderate | 210 (44.2%) | 120 (45.3%) | 18 (39.1%) | 72 (43.9%) |  |
| Poor | 123 (25.9%) | 65 (24.5%) | 16 (34.8%) | 42 (25.6%) | 0.185 |
| CPS^&^ |  |  |  |  |  |
| <1 | 142 (29.9%) | 75 (28.3%) | 18 (39.1%) | 49 (29.9%) |  |
| 1-20 | 210 (44.2%) | 120 (45.3%) | 16 (34.8%) | 74 (45.1%) |  |
| >20 | 123 (25.9%) | 70 (26.4%) | 12 (26.1%) | 41 (25.0%) | 0.087 |
| Cycle |  |  |  |  |  |
| Two | 270 (56.8%) | 156 (58.9%) | 25 (54.3%) | 89 (54.3%) |  |
| Three | 205 (43.2%) | 109 (41.1%) | 21 (45.7%) | 75 (45.7%) | 0.603 |
| cTNM |  |  |  |  |  |
| Ⅲ | 205 (43.2%) | 115 (43.4%) | 18 (39.1%) | 72 (43.9%) |  |
| Ⅳ | 270 (56.8%) | 150 (56.6%) | 28 (60.9%) | 92 (56.1%) | 0.642 |
| ypstage |  |  |  |  |  |
| yp0 | 98 (20.6%) | 80 (30.2%) | 6 (13.0%) | 12 (7.3%) |  |
| ypⅠ | 105 (22.1%) | 70 (26.4%) | 10 (21.7%) | 25 (15.2%) |  |
| ypⅡ | 130 (27.4%) | 75 (28.3%) | 12 (26.1%) | 43 (26.2%) |  |
| ypⅢ | 82 (17.3%) | 30 (11.3%) | 10 (21.7%) | 42 (25.6%) |  |
| ypⅣ | 60 (12.6%) | 10 (3.8%) | 8 (17.4%) | 42 (25.6%) | <0.001 |

* comparison among the NAIC, NAI-CTX, NAC groups;

& CPS: combined positive score;
