# Supplementary figures and images for: Anti-EGFR enhanced neoadjuvant immunotherapy versus neoadjuvant immunochemotherapy for locally advanced oral squamous cell carcinoma
Source: Front Immunol. 2025 Nov 20;16:1669368. doi: 10.3389/fimmu.2025.1669368 (PMC12675437; doi:10.3389/fimmu.2025.1669368)

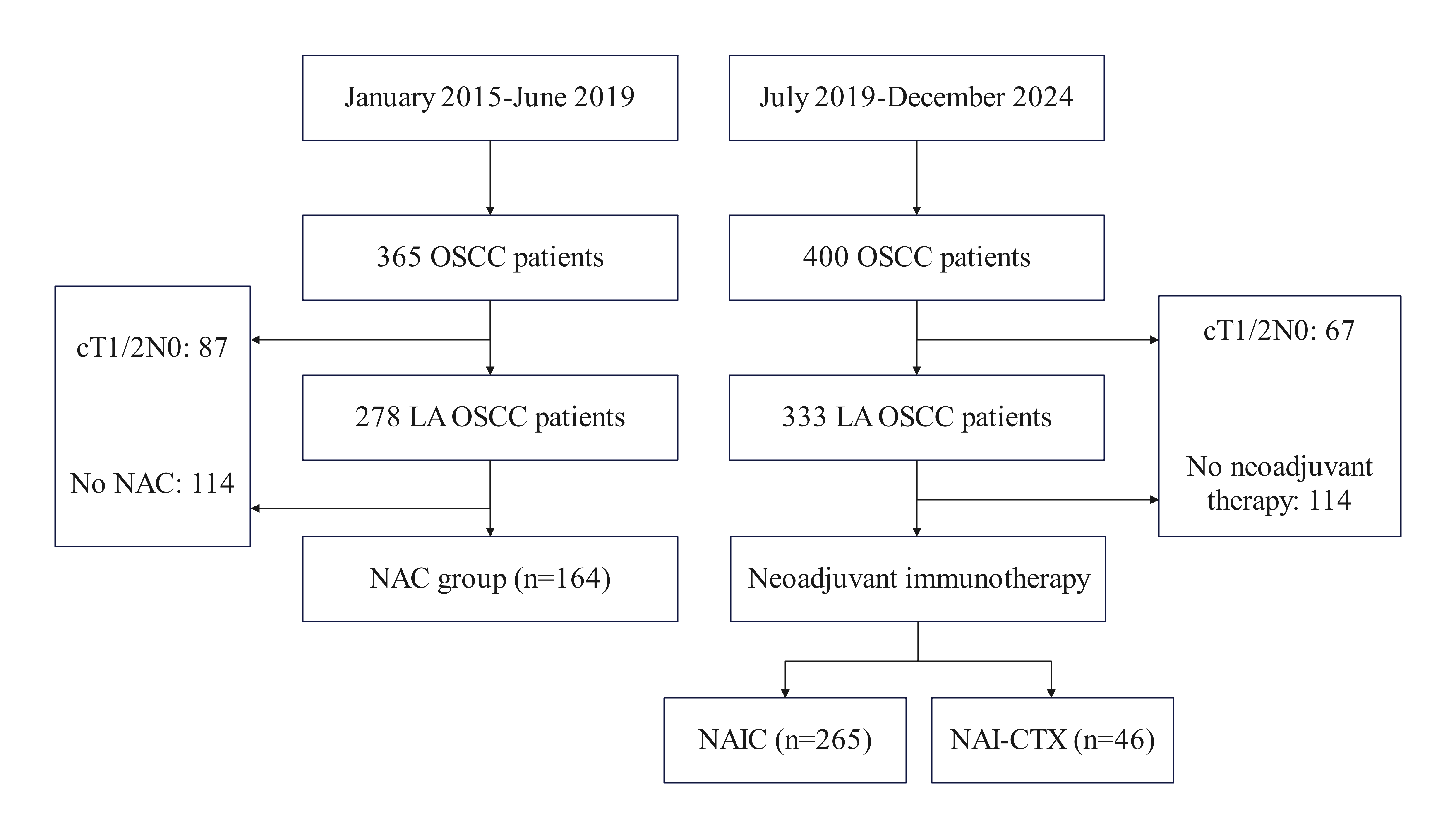

Supplement: Supplementary Figure 1 — Patient enrollment flowchart. [file Image1.jpeg]
